# *Lithraea caustic* (Litre) Extract Promotes an Antitumor Response Against B16 Melanoma

**DOI:** 10.3389/fphar.2019.01201

**Published:** 2019-10-22

**Authors:** Claudia Robles-Planells, Sofia A. Michelson, Javier Mena, Daniela Escrig, Juan L. Rojas, Giselle Sanchez-Guerrero, Ronny Hernández, Carlos Barrera-Avalos, Leonel E. Rojo, Daniela Sauma, Alexis M. Kalergis, Mónica Imarai, Ricardo Fernández, Carolina A. Robles, Elías Leiva-Salcedo, Rocio Santander, Alejandro Escobar, Claudio Acuña-Castillo

**Affiliations:** ^1^Departamento de Biología, Facultad de Química y Biología, Universidad de Santiago de Chile, Santiago, Chile; ^2^Centro de Biotecnología Acuícola, Universidad de Santiago de Chile, Santiago, Chile; ^3^Escuela de Medicina, Facultad de Ciencias Médicas, Universidad de Santiago de Chile, Santiago, Chile; ^4^Departamento de Biología, Facultad de Ciencias, Universidad de Chile, Santiago, Chile; ^5^Millennium Institute Immunology and Immunotherapy, FOCIS Center of Excellence, Facultad de Ciencias Biológicas, Facultad de Medicina, Pontificia Universidad Católica de Chile, Santiago, Chile; ^6^Departamento de Salud, Universidad de Los Lagos, Osorno, Chile; ^7^Departamento de Ciencias del Ambiente, Facultad de Química y Biología, Universidad de Santiago de Chile, Santiago, Chile; ^8^Laboratorio Biología Celular y Molecular, Instituto de Investigación en Ciencias Odontológicas, Facultad de Odontología, Universidad de Chile, Santiago, Chile

**Keywords:** cancer, topic treatment, *Lithraea caustic*, DTH response, immunotherapy

## Abstract

Melanoma immunotherapy, specifically the autotransplant of dendritic cells charged with tumors antigens, has shown promising results in clinical trials. The positive clinical effects of this therapy have been associated to increased Th17 response and delayed-type hypersensitivity (DTH) against to tumor antigens. Some synthetic compounds, such as diphenylcyclopropenone (DPCP), are capable of triggering a DTH response in cutaneous malignancies and also to induce clinically relevant effects against melanoma. In this work, we evaluated Litre extract (LExT), a standardized extract of a Chilean stinging plant, *Lithraea caustic* (Litre). As Litre plant is known to induce DTH, we used a murine B16 melanoma model to compare the topical and intratumor efficacy of LExT with synthetic DTH inducers (DPCP and 2,4-dinitrochlorobenzene [DNCB]). LExt contained mainly long chain catechols and sesquiterpenes. The intratumor injection of LExT induced a significant delay in tumor growth, similarly topical treatment of an established tumor with 0.1% LExT ointment induced a growth delay and even tumor regression in 15% of treated animals. No significant changes were observed on the T-cell populations associated to LExT treatment, and neither DNCB nor DPCP were capable to induce none of the LExT-induced antitumoral effects. Interestingly, our results indicate that LExT induces an antitumor response against melanoma in a mouse model and could bring a new –and affordable- treatment for melanoma in humans.

## Introduction

Melanoma is a type of skin cancer derived from melanocytes and is the most lethal form of malignant skin tumors. Meanwhile, melanoma shows a lower-population prevalence; its incidence has doubled in the last decade (Matthews et al., 2017); this increase has a significant impact on public health, considering that conventional therapies have limited efficacy in advanced stages of the disease. Interestingly, the treatments based on immunotherapy have shown promising results in contrast to conventional chemotherapeutic approaches (George et al., 2016). The use of high doses of IL-2 was the first anti-cancer immunotherapeutic approach, and while its use has been capable of inducing tumor remissions in patients with advanced melanoma, its effect is limited to a small fraction of patients and also associated with elevated cytotoxicity (Smith et al., 2009).

On the other hand, it has been reported that blocking antibodies against specific targets, such as the Programmed Cell Death protein 1 (PD-1) and/or against cytotoxic T-lymphocyte antigen 4 (CTLA-4), promote tumor rejection, suggesting therapeutic alternatives against this type of cancer(Curran et al., 2010). Another promising immunotherapy is the use of auto-transplant of autologous dendritic cells (DCs), which are generated from precursors cells present on peripheral blood monocytes. DCs need to be loaded with tumor antigens and then activated *in vitro*, before being auto-transplanted to the cancer patients. Previous studies on the vaccination of melanoma patients with melanoma peptides (Nestle et al., 1998; Tel et al., 2013; Schreibelt et al., 2015) or DCs cell lysate (Escobar et al., 2005; Durán-Aniotz et al., 2013; Lo et al., 2019) provided evidence of significant clinical and immunological anti-tumor responses at cancer stages III and IV. These approaches increased short-term survival and progression-free survival related to a functional tumor-specific T-cell response, with a predominant T cytotoxic(Nestle et al., 1998; Tel et al., 2013; Schreibelt et al., 2015) and helper profiles (IFNγ, Th1, and IL-17, Th17 cells) (Escobar et al., 2005; Durán-Aniotz et al., 2013; Lo et al., 2019) and a marked reduction in the proportion of CD4 transforming growth factor (TGF) β+ regulatory T cells(Lo et al., 2019). Interestingly, these immunological profiles were similar to those of delayed-type hypersensitivity response (DTH)-positive reactions against the melanoma peptides (Nestle et al., 1998) or cell lysates (Escobar et al., 2005; Durán-Aniotz et al., 2013; Lo et al., 2019), suggesting a potential role of DTH in an antitumor response against melanoma tumors.

DTH is an overreaction against a specific antigen, and it is executed in two phases. First, the sensitization phase, in which the primary contact with the antigen occurs followed by the generation of memory T cells, and second, the effector phase, in which a subsequent second-time exposure to the antigen induces macrophages attraction to the contact zone to further trigger innate and adaptive immune responses (Kobayashi et al., 2001). Allergic contact dermatitis (ACD) corresponds to a type of delayed-type hypersensitivity response to small reactive molecules (haptens), which triggers skin inflammation mediated by stimulated T cells (Nosbaum et al., 2009). ACD has been used as a tool to develop immunotherapy against cutaneous warts, based on two molecules, 2,4-dinitrochlorobenzene (DNCB) and diphenylcyclopropenone (diphencyprone; DPCP) (Holzer et al., 2006). These latter compounds have been successfully tested in the treatment of recalcitrant viral warts, alopecia, basilioma, and spinocellular carcinoma (Buckley and du Vivier, 1999). In view that DTH induction is a crucial process in those patients exhibiting an antitumor response, the induction of DTH against different forms of cancers has been clinically evaluated since 1970. As a result, DNCB has been successfully used in the treatment of cutaneous cancerous lesions, including melanoma, in which DNCB showed efficacy in reverting and decreasing metastasis (Berd et al., 2001; Von Nida and Quirk, 2003). Recent studies demonstrated that treatments with DPCP produce a inhibition of melanoma tumor growth (Damian et al., 2009), suggesting its use as a feasible option to treat melanoma cutaneous lesions ineligible/refractory to surgery (Yeung et al., 2017). There are a variety of molecules, present in insect saliva, nickel, latex, and natural compounds of plants, which have been associated to DTH responses in humans, with a high prevalence on ACD induction (Erkes and Selvan, 2014). For instance, the natural oil from the North American plant poison ivy, poison oak or poison sumac are potent DTH inductors and have been suggested to affect between 60% and 80% of the exposed population. Unfortunately, 10% to 20% of these cases display severe, dangerous responses in exposed patients. However, despite the known potential to induce strong DTH, these type molecules have limited evidence as a potential treatment against skin malignancies (Baldwin et al., 1982; Novick and Bosniak, 1986). Therefore, basic and clinical research on new DTH-inducing compounds against melanoma is interesting and newfangled.

Plants associated with DTH response produce stinging molecules from the urushiols family. For instance, *Lithraea caustica* (Litre) is an endemic Chilean tree distributed from Coquimbo (Latitude 29°57′S) to Cautin (Latitude 39°38′S) which produces high levels of an urushiol-type compound called litreol. The organics extracts of this tree induce a potent DTH response, and its direct application on cultured tumor cells induces cell death (Kalergis et al., 1997; Russo et al., 2009). However, its effect against tumors *in vivo* has not yet been evaluated. In the present study we evaluated the antitumor effect of Litre extract (LExT), a proprietary LExT of *L. caustica*. For this this aim, we used a B16 murine melanoma model. The antitumor effect of LExT was compared with those of DNCB and DPCP, two compounds previously used in human skin malignancies. LExT induced a marked delay in the growth of melanoma tumors. The antitumor effect of LExT was not associated with specific changes on T-cell infiltration, but with an increase in inflammatory damage at late stages of tumor growth. This effect of LExT is highly relevant considering that neither DNCB nor DPCP are capable of inducing a potent antitumor response in mice. The present study is, to our knowledge, the first report describing the effects of a topical formulation based on LExT as a treatment against melanoma. Given our results, we suggest that LExT could be further studied as a topic immunotherapy against melanoma in humans.

## Methods

### Tumor Cell Line Culture and Animals

Murine melanoma B16 cells were cultured in DMEM (Gibco) supplemented with 10% fetal bovine serum (FBS) (Invitrogen, Grand Island, NY) and incubated at 37°C, 5% CO_2_. Six-to-eight-weeks old C57BL/6 (H2^b^) mice were obtained from the Universidad de Santiago de Chile animal facility. Mice were fed *ad libitum*, with a 12/12-h light/dark cycle. All procedures were conducted in accord to guidelines on the recognition of pain, distress and discomfort in experimental animals described by Morton and Griffiths, except for temperature evaluation (Morton and Griffiths, 1985). Protocols were reviewed and approved by the Ethics Committee of the Universidad de Santiago de Chile. LExT was prepared by organic extraction. Briefly, fresh leaves of *L. caustica* were collected and dried at room temperature by seven days. Once dried, 40 g of leaves were mixed with 1-L petroleum ether for 20 min. After filtration, the solvent was concentrated in a rotary evaporator under vacuum until complete solvent evaporation, the extract was then recovered, and the purity was evaluated by layer chromatography using a mobile phase composed of hexane/ethyl acetate (95:5). All the reagents used were analytical grade from Merck Co.

### Sensitization and Treatment With DPCP, DNCB, and Lext

The effect of topical treatment with LExT was evaluated in mice bearing tumor previously sensitized. To do so, mice were shaved in the dorsal area and sensitized by skin application with vehicle or for each compound independently (20 µl of DPCP 2% in acetone, 20 µl of DNCB 2% in acetone or an ointment containing 0.1% LExT). Sensitization was done at 1- to 2-cm away from area where the tumor was later injected. Then, 3 days after, mice were subcutaneously injected in the lumbar zone with 100 µl of 2 × 10^6^ tumor cells/ml in PBS, with a tumor cell suspension obtained by trypsinization from cells cultured at 80% confluence. Once the tumor was detectable (0.3 mm^3^ approximately), animals were treated with the same ointment (0.1% DPCP or 0.1% DNCB or 0.1% LExT) or vehicle every other day. The effect of LExT as an intratumor treatment was also evaluated to determine the effects that mice were sensitized and then injected with tumor cells, under the same conditions aforementioned. Once a volume of approximately 2 mm^3^ has been reached, one injected dose of 50 ul of excipient or 0.1% LExT was applied to each tumor. In all cases, tumor emergence and size measurements were checked daily with a caliper. The tumor volume (mm^3^) was calculated by measuring tumor diameter with a caliper and using the expression for calculating the hemisphere volume, $V({\mathrm{mm}}^{3})=\frac{1}{12}\times \pi \times d^{3}$, where r = tumor radius; d = tumor diameter. The control group was not sensitized (non-treated). For the treatment, the mice were sensitized with an ointment containing 0.1% LExT under one armpit.

### T-Cell Population Analysis

For both treatment protocols, the end-point criterion was when the tumor volume reached 250 mm^3^ or a maximum period of 60 days without tumor. Afterward, mice were sacrificed by cervical dislocation, and the spleen and the tumor were removed. Spleen was disaggregated using a stainless-steel mesh (100 µm), erythrocytes were removed by differential lysis using an ACK buffer (NH_4_Cl 155 mM, KHCO_3_ 10 mM, Na_2_EDTA 1 mM, pH 7.3) with gentle agitation for 5 min (Leiva-Salcedo et al., 2011), then centrifuged at 1200*g* for 10 min and discarded the supernatant. In C57BL/6 wild-type mice, splenocytes were suspended at 2 × 10^6^ cells/ml in 1 ml of cold blocking buffer (2% FBS in PBS, IF buffer) and incubated at 4°C per 30 min. Cells were stained with antibodies against the cell surface markers CD4, CD8, and CD25, with anti-mouse CD4-FITC (eBioscience), anti-mouse CD8-PE (eBioscience), and anti-mouse CD25-PE (eBioscience), respectively. Then resuspended in a fixation/permeabilization solution (Fix/Perm; eBioscience) and incubated with anti-Foxp3-PerCP (eBioscience) antibody for Treg population, anti-human/mouse RORγ(t)-PE (eBioscience) antibody for Th17 population and anti-human/mouse T-bet, PerCP-Cy5.5 (eBioscience) antibody for Th1 population, simultaneously to anti-CD4-FITC antibody labeling (eBioscience, USA). All samples were analyzed by flow cytometry using a BD Accuri C6 cytometer (BD Bioscience, San Jose, CA), and data were analyzed by FlowJo 7.6.1 software (Tree Star, Inc.). For details on these methods, please see Morales et al. (2017).

### Histopathological Procedures

Tumors were removed and fixed in 10% buffered formalin for 24 h and then dehydrated with an increasing sequential concentration of ethanol (Histoprocesser, Leica ASP300). Then tumors were included in paraffin and cut at 5-µm thick with a rotatory microtome (Leica RM 2235). The samples were stained with hematoxylin-eosin (HE) using a tissue stainer (Leica ST5020). Histological samples were observed in a light microscope (Olympus CX41) coupled with a digital camera and using an achromatic wide field objective. Images were acquired at 4×, 10×, 40×, and 100×. Ten fields were examined per sample to evaluate necrosis, hemorrhage, inflammatory infiltrate, vascularization, and the level of tissue perfusion (expressed as the number of blood vessels). In all samples, we defined absence (−), low (+), high (++), and extremely high level (+++). The levels of necrosis indicate the destruction of the neoplastic cells in response to a direct cytotoxic action mediated by the inflammatory infiltration. Neoplastic cells viability was expressed as melanin levels (i.e., low melanin, low cell viability). The less populated tissue areas containing a high accumulation of cell debris were defined as necrosis. The hemorrhage was defined as the interstitial tissue areas with erythrocytes extravasation. The inflammatory infiltration was characterized by the presence of leucocytes, monocytes, and polymorph nuclear cells. The vascularization was defined as the number of capillary vessels present in the samples. Tumor cell viability was determined by the intensity of nuclear chromaticism, the presence of defined cell limits, and melanin production.

### Chemical Analysis

A Thermo Scientific GC–MS system (GC: model: Trace 1300 and MS: model TSQ8000Evo) was used to analyze the sample. The separation was performed on a 60 m× 0.25 mm internal diameter fused silica capillary column coated with 0.25-μm film DB-5MS. The injector and the detector temperatures were, respectively, 200°C and 250°C. Oven temperature was held at 40°C for 5 min, then programmed from 40°C to 300°C at 5°C/min and finally maintained at 300°C for 90 min. The mode used was splitless injection; helium was used as carrier gas; and flow rate was 1.3 ml/min. Mass spectra were recorded over a range of 40- to 400 atomic mass units at 0.2 s/scan. Solvent cut time was 11 min. Ionization energy was 70 eV. The chemical composition of the oil was identified by comparing its spectra with those of a NIST14 library and confirmed by contrasting their retention indices with data published in other studies.

### Statistics

Tumor onset was ere analyzed using Kaplan–Meir curves and the log-rank test. Tumor-onset growth and size were analyzed by Kruskal–Wallis one-way ANOVA, two-way ANOVA followed by the Sidak post-test, and linear regression fitting and comparison. Th1, Th17, and Treg subpopulations were analyzed by the Mann–Whitney test. Analyses and graphs were performed using GraphPad Prism 5.01 software. Results are expressed as means ± standard error of means (SEMs). Statistical differences were considered significant when *p* < 0.05.

## Results

**Figure 1:**
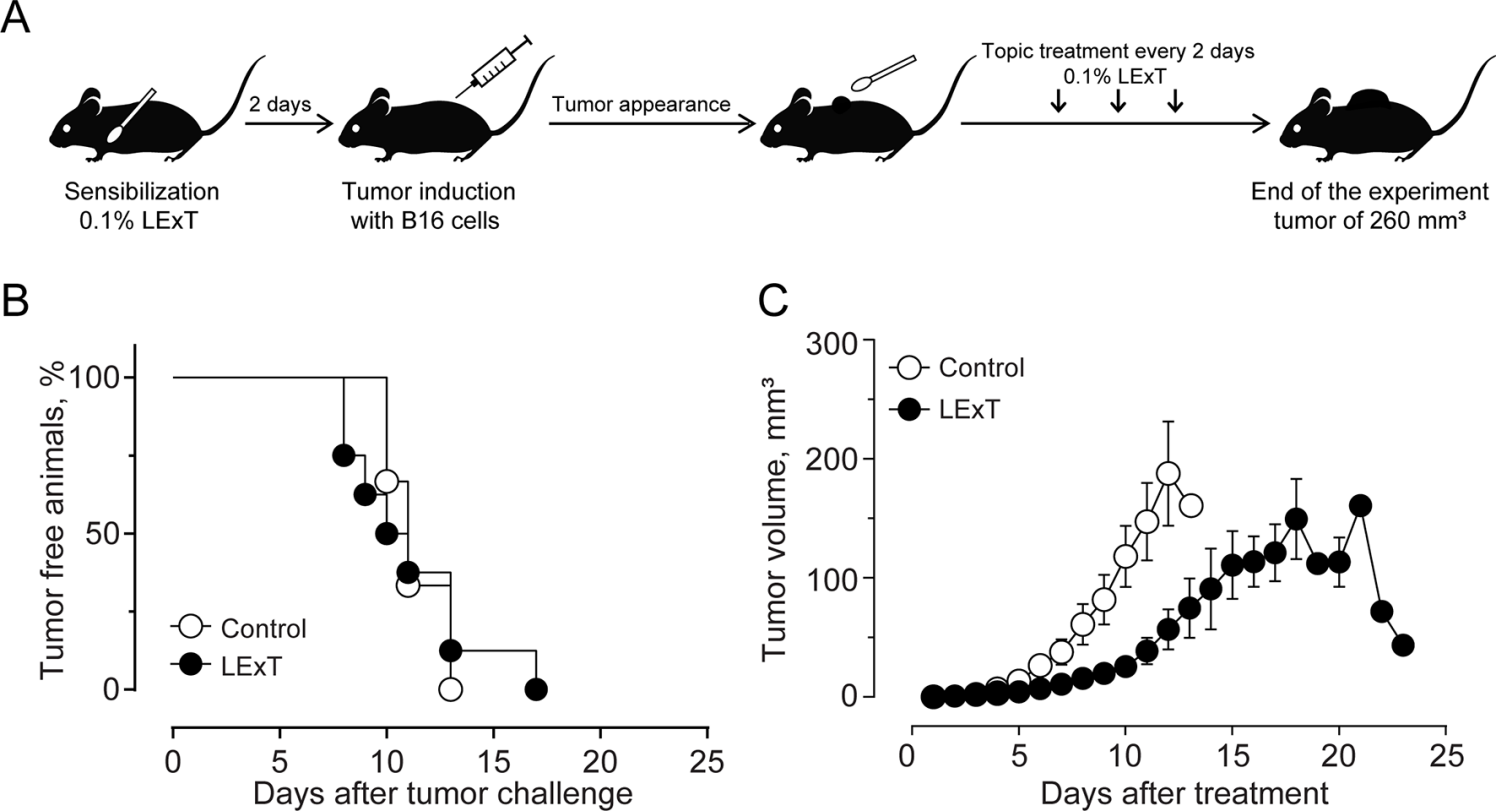
Effect of Litre extract (LExT) as a topical treatment on tumor volume in C57BL/6 mice. **(A)** Treatment diagram. Animals were sensitized and then topically treated with an excipient or 0.1% LExT every two days until the end of the experiment; **(B)** Kaplan-Meier analysis for tumor onset and **(C)** Kinetics of tumor growth on control excipient-treated and LExT-treated mice. The abrupt decrease in tumor volume at day 21 in the LExT group indicates a tumor regression in 17% of treated mice. Values are mean ± SEM of 8-12 animals per group.

**Figure 2:**
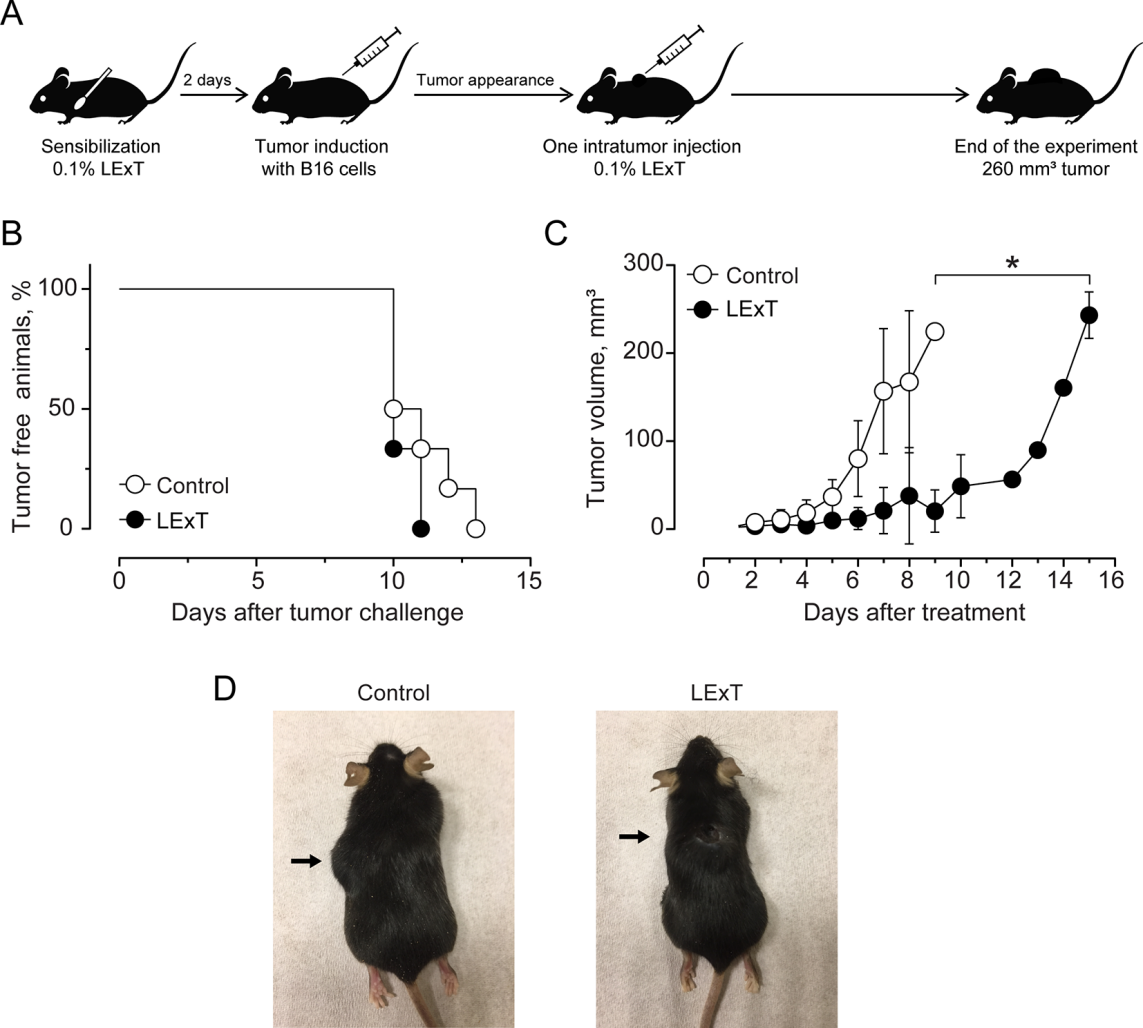
Effect of Litre extract (LExT) intra-tumor treatment in tumor volume in C57BL/6 mice. **(A)** Treatment diagram. Animals were sensitized and then intratumorally injected with one dose of 50 μL of excipient or 0.1% LExT; **(B)** Kaplan-Meier analysis for tumor onset and **(C)** Kinetics of tumor growth on excipient-treated (control) and LExT-treated mice; Values are mean ± SEM of 8 animals per group. **(D)** Representative images of the tumor in LExT-treated and non-treated animals at the end of the experiment. Values are mean ± SEM of 8-12 animals per group. *p<0.05, Assessed by Kruskal–Wallis one-way ANOVA.

The use of epifocal DTH inductors against unresectable cutaneous melanoma lesions has reached clinical relevance, as they are efficacious chemotherapies for the type of malignancies. In this work, we evaluated the effect of LExT, an urushiols-containing extract from Litre described has a potent DTH inducer on a mouse (López et al., 1998), in a model of melanoma. Catechol analysis was performed as described elsewhere (Urzúa et al, 2011) and the results are shown in **Table 1**. Our first approach was to evaluate whether LExT induces *in vivo* antitumor response. For this purpose, after a sensibilization phase, we intratumorally applied either vehicle or LExT to animals when the tumor size reached 2 mm^3^ average (**Figure 1A**), which normally occurred between days 10 and 13 after the injection of tumor B16 cells (**Figure 1B**). We observed that it took 4 days for the mice intratumorally injected with a vehicle to show significant tumor growth, and maximal tumor volume was reached at day 9 after vehicle treatment (**Figure 1C**). Whereas in the LExT-treated animals, it took 8 days after treatment to reach significant tumor growth, and the maximal volume was reached at day 15 after LExT treatment. The average tumor volume in LExT-treated animals was significantly lower than that of vehicle-treated counterparts at day 5 after challenge (**Figure 1C**). These data demonstrated the robust inhibitory effect of LExT on tumor development. Our next aim was to evaluate whether the epifocal application of LExT would induce similar results as the intratumor injected LExT therapy (**Figure 2A**), because epifocal therapies are significantly less toxic than systemic therapies. Animals were challenged with alive tumor cells, once the tumor was established, the animals received topical treatment with either placebo or 0.1% LExT ointment every other day (**Figure 2A**). Tumors were detectable between 8 and 17 days after tumor cells challenge, then the animals were grouped to avoid differences in tumor size among both groups (Control and LExT-treated mice). Placebo-treated animals showed an increase in the tumor volume from day 5, reaching a maximum tumor volume at day 12 (**Figure 2B**). Similar to intratumor treatment the epifocal treatment induced a delay in the time to maximal tumor growth, as in the Control group it took 11 to 12 days to reach the maximal volume, while the LExT-treated animals reached this stage at 18-20 day. Also, in the LExT-treated group, 17% of tumor regression was observed (**Figure 2C**). Altogether these results suggest that LExT (intratumor or topical) has a robust antitumor effect in melanoma tumors.

**Table 1:** Identity of the chemical compounds of LExT identified by GC/ MS/MS.

N°	Compound	RI_exp_	Relative abundance (%)	Identification
1	Caryophyllene	1436.73	0.68	RI, MS
2	Aromandendrene	1463.90	0.17	RI, MS
3	Humulene	1467.29	0.62	RI, MS
4	γ-Muurolene	1487.00	0.06	RI, MS
5	β-Ionone	1493.52	0.11	RI, MS
6	α-Muurolene	1512.03	0.14	RI, MS
7	Cuparene	1523.96	0.19	RI, MS
8	γ-Muurolene	1528.62	0.12	RI, MS
9	β-Cadinene	1535.37	0.15	RI, MS
10	Calamenene	1537.47	0.12	RI, MS
11	Dihydroactinidiolide	1552.52	0.34	RI, MS
12	α-Calacorene	1559.58	0.12	RI, MS
13	Caryophyllene oxide	1605.44	1.08	RI, MS
14	Humulene-1,2-epoxide	1632.83	1.56	RI, MS
15	Cadalene	1693.15	0.35	RI, MS
16	Neophytadiene	1833.88	0.72	RI, MS
17	Perhydrofarnesyl acetone	1841.28	0.34	RI, MS
18	Phytol	2110.86	5.12	RI, MS
19	4,8,12,16-Tetramethylheptadecan-4-olide	2360.75	0.44	RI, MS
20	Ginkgol	2493.72	2.79	RI, MS
21	Tetracosanal	2629.24	1.87	RI, MS
22	3-(Pentadec-10-enyl)-catechol	2678.84	12.48	RI, MS
23	Hexacosanal	2832.25	4.41	RI, MS
24	1-Hexacosanol	2895.97	12.52	RI, MS
25	Octacosanal	3036.18	6.35	RI, MS
26	Octacosanol	3098.95	7.09	RI, MS
27	γ-Sitosterol	3366.40	7.61	RI, MS
	**Total sesquiterpenes**	**5.8%**		
	**Long chain catechols**	**9.6%**		
	**Total catechols**	**22.1%**		

**Figure 3:**
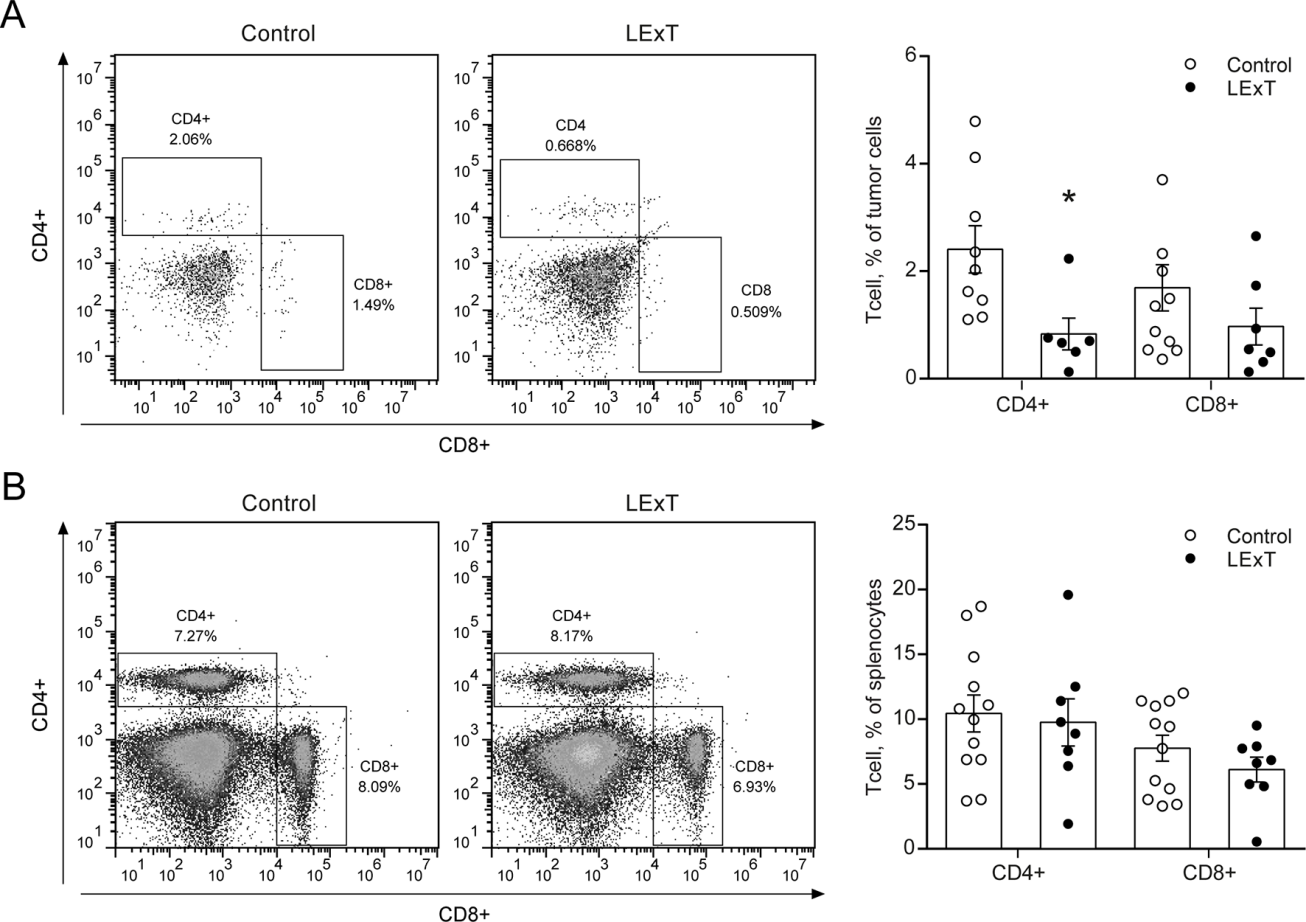
Effect of topical Litre extract (LExT) treatment in splenic and tumor-infiltrating CD4+ and CD8+ T cells in C57BL/6 mice. Representative dot plots (left panel) and quantification of CD4^+^ and CD8^+^ T cells identified in **(A)** total tumor-infiltrating and **(B)** splenocytes cells excised from LExT-treated and non-treated mice. Values are mean ± SEM, *p<0.05 *vs.* excipients, assessed by the Mann–Whitney test.

**Figure 4:**
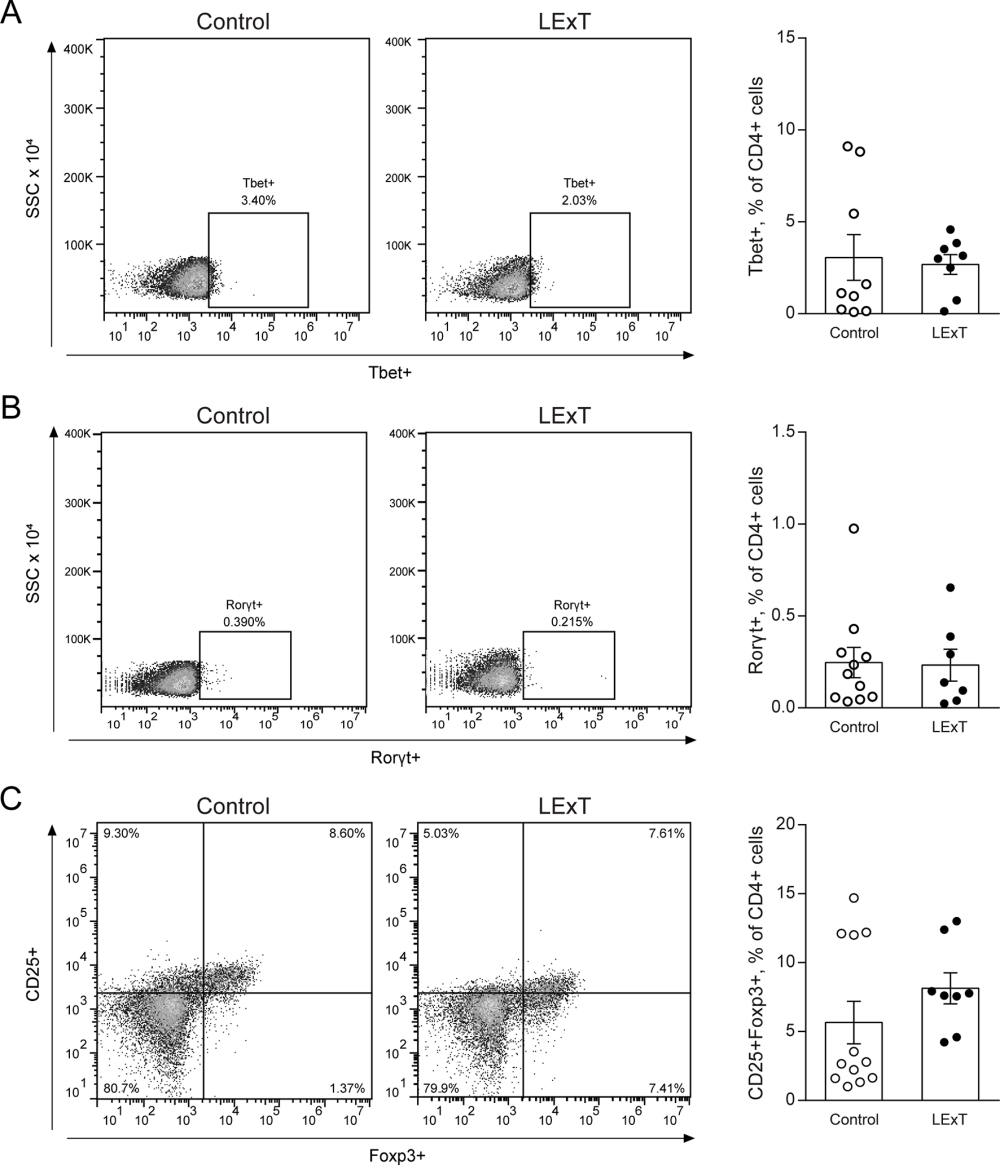
Effects of Litre extract (LExT) as a topical treatment on spleen levels of Th1, Th17, and T regulatory (Treg) cells in C57BL/6 mice. Representative dot plots (left panel) and quantification of **(A)** Th1 (CD4+ Tbet+), **(B)** Th17 (CD4+ RORγ(t)+) and **(C)** Treg cells (CD4+ CD25+ FoxP3+) in LExT-treated and non-treated mice. Values are Means ± SEMs.

To further characterize the LExT mode of action, we analyzed whether LExT induces changes of T-cells populations and subpopulations that could suggest an immune response against the tumor. This effect was evaluated both in the tumor and in the spleen at the end of the tumor growth period. We detected a significant decrease in CD4+ tumor infiltrated cells, without significant changes in CD8+ cells (**Figure 3A**) compared with controls, whereas in the spleen, neither CD4+ nor CD8+ showed any changes respect to controls (**Figure 3B**). Similarly, LExT treatment did not induce significant changes in CD4+ subpopulation expressing markers for Th1, T-bet+CD4+ (**Figure 4A**), Th17, RORγ(τ)+CD4+ T cells (**Figure 4B**) nor in Treg, Foxp3-CD25+CD4+ (**Figure 4C**) positive cells in the spleen. These results suggest at the in our experimental conditions, no significant changes in populations and subpopulations of T cells were observed in response to LExT, except for a significant decrease in CD4+ infiltrating cells.

**Figure 5:**
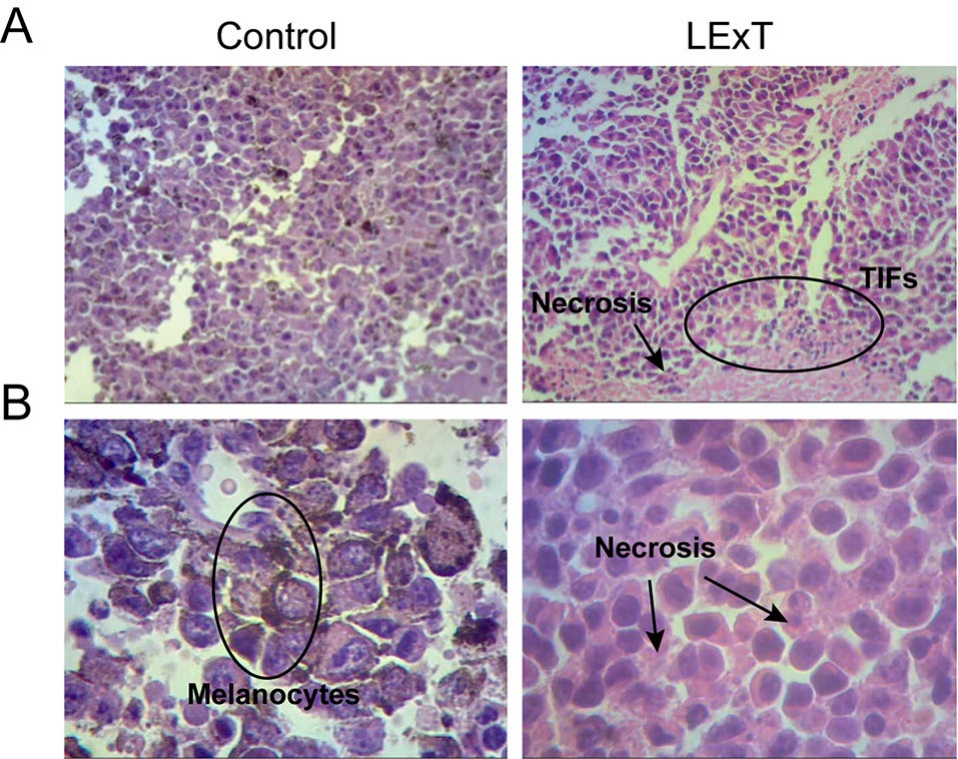
Histological characterization of tumors derived from Litre extract (LExT)-treated C56BL/6 mice. Representative images of tumor obtained from Control-treated (left column) and LExT-treated (right column) mice. **(A)** and **(B)** show representative images for 5X and 40X magnifications, respectively. Each image is representative of 7-10 tumors obtained from each treatment. TIFs, tumor-infiltrating lymphocytes.

Although changes on CD4+ and CD8+ T-cell populations were not observed in response to LExT in our model of melanoma, the fact that *L. caustic* is known to induce inflammation led us to evaluate the presence of inflammatory markers in the tumors of LExT-treated animals. To do so, we studied histological parameters of focal necrosis, and inflammatory leukocytes infiltrate inside the tumors. We found that more inflammatory cells infiltrates in LExT-treated tumors (right column) than in the tumors of vehicle-treated animals (**Figure 5**) as shown in 5× (**Figure 5A**) and 40× (**Figure 5B**) images (**Table 2**). We also found evidence of necrosis in tumors of LExT-treated animals. These results indicate that LExT induced a marked inflammation in the tumors.

**Table 2:** Quantification of tumor histopathological features from control and LExT-treated mice.

	Control			LExT		
	low	high		low	high	
**Necrosis**	5	10	ns	1	8	*
**Inflammation**	4	11	ns	3	6	ns
**Vascularization**	4	8	ns	5	4	ns
**Melanin**	7	8	ns	7	2	*

ns: non-significant difference, Low: mild response, High: severe response, *p < 0.05, assessed by Kruskal–Wallis one-way ANOVA.

**Figure 6:**
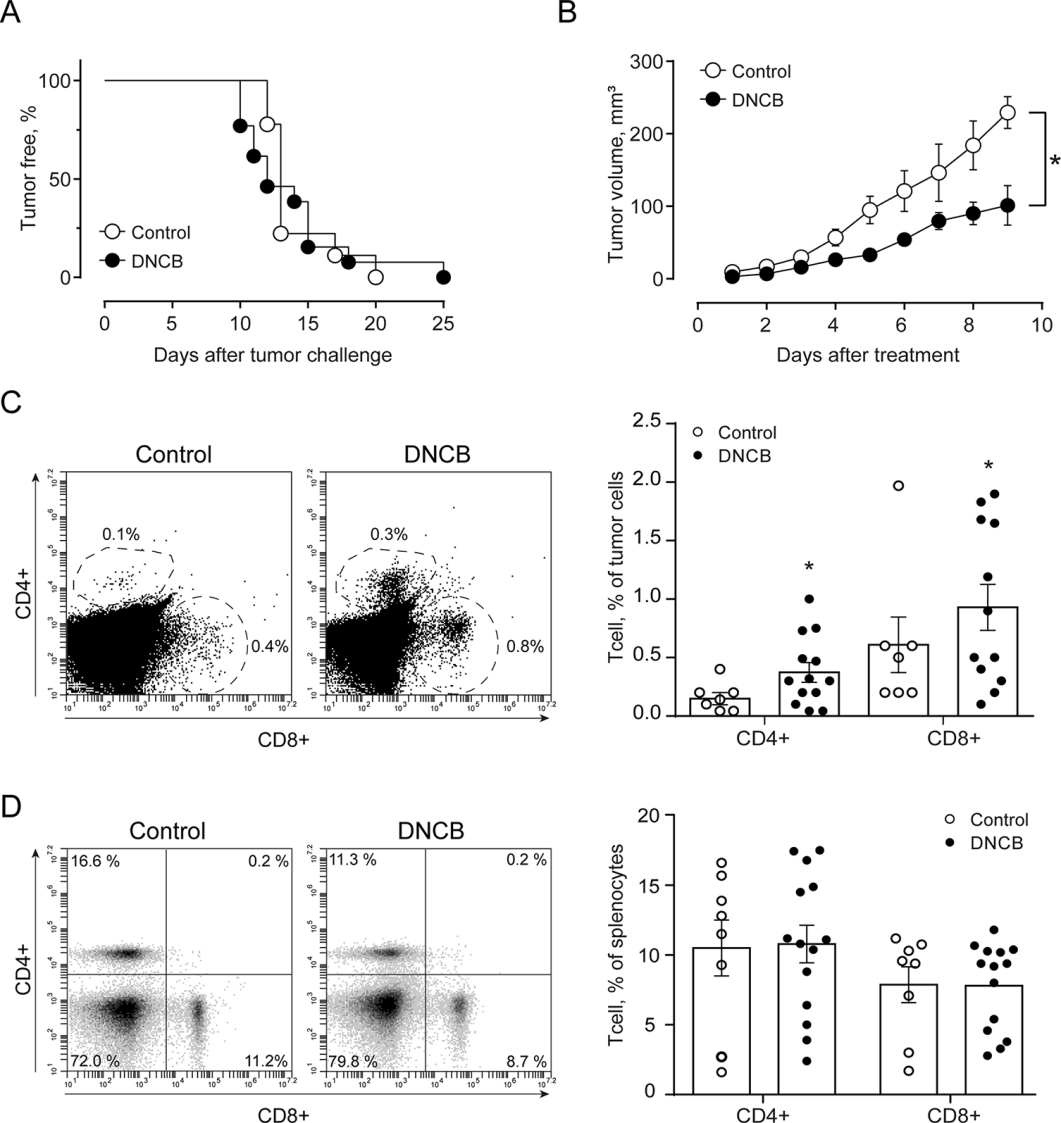
Effect of DNCB treatment on B16 tumor onset and growth (volume) and CD4+ and CD8+ cells in the tumor and spleen in C57BL/6 mice. **(A)** Kaplan-Meier analysis for tumor onset on excipient-treated (control) and DNCB-treated mice; **(B)** Kinetics of tumor growth in DNCB-treated and non-treated animals. * *p* < 0.05, assessed by Kruskal–Wallis one-way ANOVA. Representative dot plots of CD4^+^ and CD8^+^ cells identified in **(C)** total tumor-infiltrating cell and **(D)** splenocytes cells excised from DNCB-treated and non-treated mice. Values are mean ± SEM, *p < 0.0 vs. excipients, assessed by the Mann–Whitney test.

**Figure 7:**
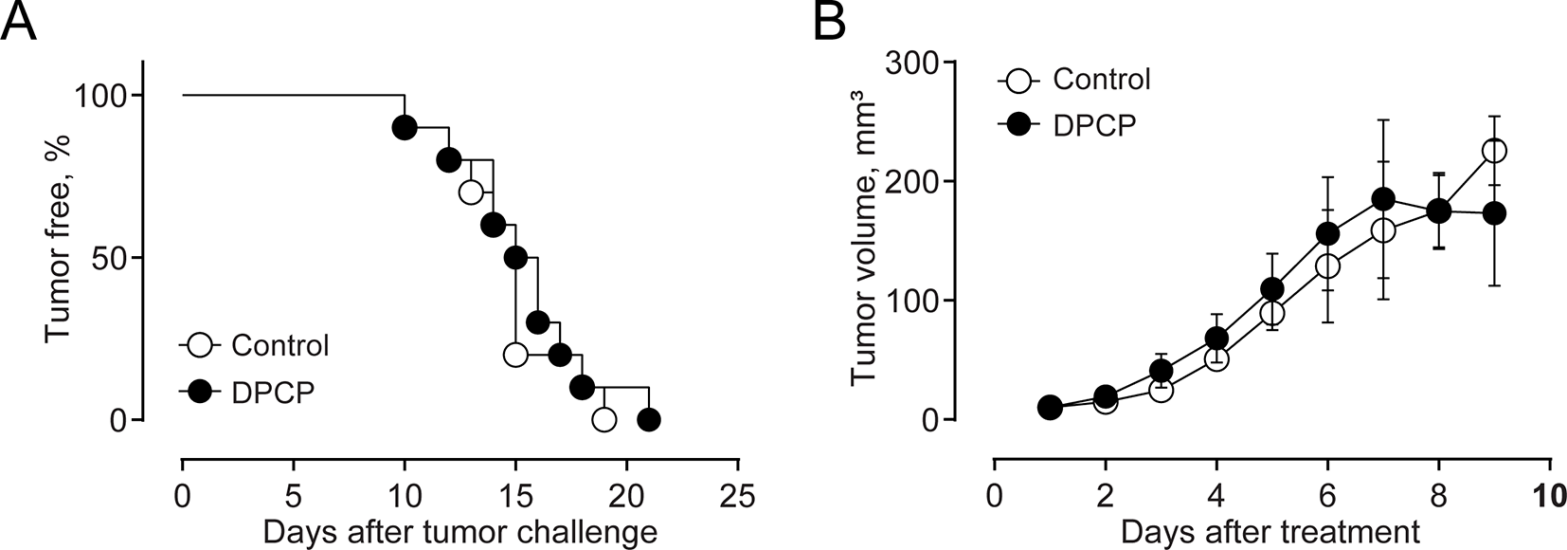
Effect of DPCP treatment on B16 tumor onset and growth (volume) and CD4+ and CD8+ cells in the tumor and spleen in C57BL/6 mice. **(A)** Kaplan-Meier analysis for tumor onset on excipient-treated (control) and DPCP-treated mice; **(B)** Kinetics of tumor growth in DPCP-treated and non-treated animals. Values are Means ± SEMs.

Finally, we compared the effects elicited by epifocal LExT against melanoma with those of clinically validated contact sensitizers (DNCB, DPCP) previously used as an epifocal treatment in the same skin cancer. Initially, we evaluated the effect of the DNCB treatment, a well-studied DTH inducer (Wack et al., 2002). To evaluate the effects of DNCB on tumor growth, we set a tumor on C57/BL6 mice, injecting tumor cells, as described in the *Methods* section of this work. Mice developed detectable tumors between 10 and 21 days post-tumor cell challenge (**Figure 6A**). In agreement with previous reports, systemic DNCB induced a mild inhibitory effect on tumor growth (**Figure 6B**), associated with an increase in CD4+ and CD8+ T-cell levels in the tumor infiltrates (**Figure 6C**), without significant changes in these population in the spleen (**Figure 6D**). These results suggest that increases in the specific T-cell population in the tumor might be responsible for the delay in tumor growth for DNCB. We further evaluated the current gold standard hapten used to treat melanoma, DPCP (Wack et al., 2002; Damian et al., 2013; Yeung et al., 2017). Strikingly, DPCP treatment did not induce antitumor effects in our murine model of melanoma (**Figure 7**), nor any change in conventional populations of cells in the spleen or the tumor tissue (data not shown). Altogether, these results indicate that LexT induces an antitumoral response and deserves further investigations to develop a new anti-melanoma immunotherapy expanding the currently limited therapeutic options for this disease.

## Discussion

In this study, we reported that epifocal treatment with LExT decreases melanoma tumor growth in B16 mice model. Intriguingly, we observed that the gold standard epifocal treatments used against melanoma in humans, DNCB and DPCP, were not effective against melanoma in mice, as only DNCB was able to induce a mild antitumor effect in our murine model of melanoma.

DNCB and DPCP are the most commonly used compounds in immunotherapy against recalcitrant viral warts, basilioma and spinocellular carcinoma. Unfortunately, DNCB has shown mutagenic effects in the “Ames Test.” Although no clinical evidence has yet shown carcinogenesis susceptibility in patients treated with DNCB (Buckley and du Vivier, 1999), this potential mutagenic toxicity has restricted DNCB use in humans. Current evidence suggests that DPCP has been characterized as an efficacious antitumor contact sensitizer with no mutagenic risk, opening a new avenue for anticancer therapeutic strategies based on DTH induction (Singh and Lavanya, 2010). On this regard, both DPCP and DNCB, have been used in the treatment of several cutaneous lesions in humans (Von Nida and Quirk, 2003; Damian et al., 2009; Harper et al., 2014; Trowbridge et al., 2014). Interestingly, no studies are describing comparable effects of these drugs in mouse models of melanoma. Probably, the differences observed comparing both models can be explained by different experimental designs, particularly in the duration of the treatments. In murine experiments, for example, the treatments typically last for three weeks, whereas the human treatments last up to 18 weeks (Damian et al., 2013; Yeung et al., 2017). On this regard, Sutmuller et al. inoculated low amounts of B16 cells (two orders of magnitude lower), it allowed them to follow up the tumor emergence for up to 12 weeks (Sutmuller et al., 2001). This strategy seemingly offers the possibility to improve the comparisons by increasing the duration of the treatments for up to 10 weeks.

Further research on the potential side effects of LExT is necessary to evaluate its safety as an anticancer therapy since aerial parts of *L. caustic* is known to induce allergic reactions, dermatitis, and skin inflammation including in mice (López et al., 1998). Unfortunately, there are no reliable epidemiological data on the prevalence of the Litre-induced DTH, or even more important, the percentage of the population that displays highly severe reactions when they come in contact with urushiols-congaing plants. An estimation based on the statistics for other urushiol-containing plants suggests that the population sensitive to Litre would be around 75% to 90%. Unlike other urushiols, litreol’s effect was studied on cancer cells viability, which demonstrated a direct impact on cells viability (Russo et al., 2009). Urushiols are lipid-soluble immunogenic molecules, composed by a catechol nucleus substituted on C4 with a C15 hydrophobic chain, in the case of poison ivy, or with a C17 hydrophobic chain, in poison oak (Kalish et al., 1994), or substituted on C3 with a C15 hydrophobic chain, in the case of litreol (Russo et al., 2009). Several mechanisms by which urushiols act as immunogens have been suggested, such as intracellular hapten processed by the endogenous pathway to be presented to CD8+ T cells, extracellular haptens processed by the exogenous way to activate CD4+ T cells; and finally, urushiol could be also recognized by CD8+ T cells without processing, possibly by direct conjugation with class I MHC (Kalish et al., 1994).

Regarding the latter suggested urushiol-mediated immunogenic mechanisms, isolated CD8+ T cells from skin lesions of urushiol sensitized human subjects favor their role in the effector phase of contact dermatitis produced by poison ivy (Kalish and Johnson, 1990). Current studies indicate that IL-4 is an important downregulator during the elicitation phase of contact sensitivity (CS), demonstrated by the presence of high levels of IL-4 mRNA in the dermis and epidermis following skin challenge with trinitrochlorobenzene (TNCB) (Asada et al., 1997; Arakawa et al., 2018). Also, mice treated with anti-IL-4 mAb shortly before ear challenge showed increased ear swelling responses, this being accompanied by enhanced IFNγ, IL-2, and IL-12 mRNA signals in the dermis. These findings suggest that IL-4 blunts CS by regulating local production of proinflammatory cytokines (Asada et al., 1997), which could explain the increase in inflammation detected in our histological sections of melanoma tumors. The hapten urushiol can bind to epidermal cells, both keratinocytes and Langerhans cells, which may then migrate through lymphatics vessels to lymph nodes, where they present the antigen to T cells, allowing the recruitment of immune cell to the tumor area. Another feasible alternative is that the keratinocytes may have an initial recognition of the antigen that produces its activation, which then causes the recruitment of leukocytes and production of cytokines, including IL-1, IL-6, and GM-CSF (Kalish, 1990). This way, keratinocytes would participate in antigen presentation process, as well as in T-cell activation. The close relationship between melanocytes and keratinocytes may play a key role in the anti-tumor response mediated by litreol treatment, because the keratinocytes can mediate melanocyte functions via several pathways, including cell-cell adhesion, cell-matrix adhesion, and paracrine signaling (Chung et al., 2011).

While we did not corroborate the described effects of *Litre* on the induction of DTH response (López et al., 1998), we cannot rule out the participation of DTH response in the effect of LExT against melanoma. In our experiments, the mode of action for the efficacious anticancer response of LExT remain to be elucidated, specifically the molecular mechanisms of the immune response. Altogether it is feasible to propose that a LExT-induced DTH (López et al., 1998) response could partially explain the antitumor we have observed. The fact that IL-17–mediated DTH is crucial for antitumor response in melanoma supports this idea (Escobar et al., 2005). On this regard, Muranski et al. described that adoptive transfer of Th17 lymphocytes in mice challenged with B16 cells generate a complete regression of the tumor, inducing up to 100% survival after 60 days post tumor challenge (Muranski et al., 2008). Intriguingly, in our study, T cells positive for Th1 and Th17 markers at a systemic level did not show measurable changes at the end of the experiment. A possible explanation for this result is that changes in the levels of Th1- and Th17-positive cells occurred during the early stages of the tumor development in animals treated with LExT.

Meanwhile, in late stages, the increased tumor mass at the end of the experiment could mask possible changes on T-cell population. Even more, at the end of the treatment, the tumor could have overcome the immunological restrictions occurred during the equilibrium phase, leading to fully developed tumor (Finn, 2012). For the litreol specifically, Lopez et al. demonstrated that the contact dermatitis induced by litreol has two components: a primary T CD4+ cell independent and a secondary CD8+ T dependent and regulated by CD4+ T cells (López et al., 1998). Regardless, while no significant changes or apparently inconclusive changes (associated to a decrease of CD4+ cells) were observed in our experiments, tumor-infiltrating specific CD4+ and CD8+ T-cell population could be a small fraction of the total T cells. To characterize whether our treatment induces a specific antitumor response, and to describe the role of T-cell subpopulations on the antitumor mediated effects, it is necessary to perform new experiments, e.g., using the B16-OVA cell model that will allow the study of specific OVA T-cell induction, among other parameters. Similarly, the exact role of the subpopulations and the immune mechanism remain to be elucidated.

Finally, we conclude that easy-to-use contact allergens applied topically, such as LExT, provide advantages over other therapies requiring specialized equipment and laboratory facilities to generate vaccines for cancer. In some cases, only the sensitization without direct challenge over the tumor was enough to achieve the antitumor efficacy of a complete treatment with LExT. Our results on the inhibition of tumor development suggest that LExT could be of great help in the development of new therapies for local melanoma.

## Author Contributions

SM and CR-P: Both authors equally contributed to this work, CR performed the LExT preparation, DE, AE, JM, DS, JR, MI: Performed DPCP and DNCB experiments, SM, GS-G, CB-A, RH CR-P, DS: performed LExT experiments, CR-P, CB-A, RF, EL-S, DS, LR, AK, MI, CA-C participated in experimental design, outlined and wrote the manuscript, RS: performed LExT GC/MS//MS analyses.

## Funding

This work was funded by FONDECYT 1110734 and 1161015 (MI), 11140731 (EL-S) PAI 79140059 (EL-S), 11140915 (LR), 1180666 (AE), DICYT and DGT s/n (CA-C), CONICYT fellowship (CR-P, CB-A), CONICYT FONDEQUIP/GC MS/MS EQM 150084.

## Conflict of Interest

The authors declare that the research was conducted in the absence of any commercial or financial relationships that could be construed as a potential conflict of interest.

## Abbreviations

CD, Cluster of differentiation; FOXp3, Forkhead box p3; Tbet, T-box expressed in T-cells; RORγ(t), RAR-related orphan receptor gamma; DPCP, Diphenylcyclopropenone; DNCB, Dinitrochlorobencene; LexT, Litre Extract; DTH, Delayed-type hypersensitivity; Th, Lymphocyte T helper; CTLA, Cytotoxic T Lymphocyte-Associated; APC, Antigen presenting cell; DC, Dendritic cell; ACD, Allergy contact dermatitis.
